# Periprosthetic total knee fracture after remote reconstruction of the anterior cruciate ligament: a case report

**DOI:** 10.1186/s13256-017-1448-3

**Published:** 2017-09-29

**Authors:** Sahil Kooner, Eric Gibson, Marcia Clark

**Affiliations:** Department of Orthopedics, University of Calgary Cumming School of Medicine, 3330 Hospital Drive NW, Calgary, Alberta T2N 1N4 Canada

**Keywords:** Periprosthetic, Total knee arthroplasty (TKA), Anterior cruciate ligament (ACL) reconstruction, Stress riser effect, Distal femur fracture

## Abstract

**Background:**

Distal femoral fracture is a rare, but significant, postoperative complication of anterior cruciate ligament reconstruction. However, there has not been a reported case of periprosthetic total knee arthroplasty fracture associated with a previous anterior cruciate ligament repair.

**Case presentation:**

We report the case of a 51-year-old white man with a history of total knee arthroplasty and remote anterior cruciate ligament reconstruction, who presented with a distal femoral periprosthetic fracture at the site of a previous anterior cruciate ligament augmentation staple.

**Conclusions:**

Based on these findings, it may be important to consider removal of previous anterior cruciate ligament hardware prior to total knee arthroplasty to reduce risk of periprosthetic fracture, which should be determined on a patient-specific basis.

## Background

Total knee arthroplasty (TKA) and anterior cruciate ligament (ACL) reconstruction are among two of the most common orthopedic procedures performed each year, accounting for approximately 700,000 combined procedures annually in the United States of America (USA) alone [1, 2]. Patients requiring ACL reconstructions typically present at a younger age and are more likely to partake in riskier athletic activities [2]. As such, this population is at higher risk of developing early osteoarthritis (OA) due to several mechanisms, including increased loading, repetitive trauma, and abnormal knee kinematics post-ACL rupture [3]. Given these risk factors, it is no surprise that a number of patients with ligament reconstructions go on to require TKA for treatment of arthritic pain.

With a larger number of people requiring both ACL repairs and TKA, the complexity of primary TKAs in this population increases as well. With increased complexity, also comes greater risk of complications, including periprosthetic knee fractures. These fractures can be due to a number of biomechanical factors, but rarely is any thought given to the effect of previous ACL reconstructions. In fact, femur fracture post-ACL reconstruction is an extremely rare complication [4].

Our literature search identified 19 case studies describing distal femur fractures after ACL reconstruction; however, none of the patients in these studies had a TKA prosthesis at the time of fracture [4–21]. As far as the authors of the study are aware, there are no reported cases in the literature describing periprosthetic TKA fractures associated with the effects of a previous ACL reconstruction.

## Case presentation

**Fig. 1:**
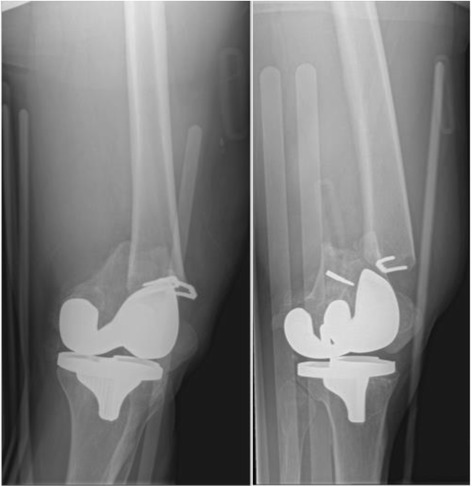
Anteroposterior and lateral view of left femoral periprosthetic fracture prior to reduction

**Fig. 2:**
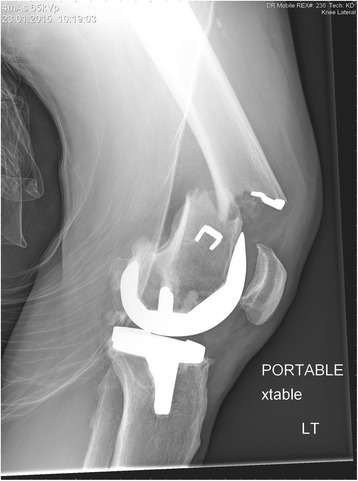
Lateral view of left femoral periprosthetic fracture prior to reduction

A 51-year-old white man presented with left thigh pain and inability to bear weight after a ground level fall. This was an isolated injury, which he described as a twisting mechanism with his knee contacting the floor after slipping. Prior to this injury, he was independently ambulant. His past medical history was significant for type-1 diabetes mellitus, retinopathy, and hypertension. His past surgical history was significant for a left corneal transplant 10 years prior, as well as a successful cruciate-retaining TKA 8 years prior. He also had remote history of ACL reconstruction in his twenties; unfortunately previous operative details for this procedure were not available. Radiographic examination revealed a left distal femoral periprosthetic fracture with a well-seated and well-aligned cruciate-retaining implant. A short oblique supracondylar fracture line originated distally from one of his previous ACL ligament augmentation staples (Figs. 1 and 2).

**Fig. 3:**
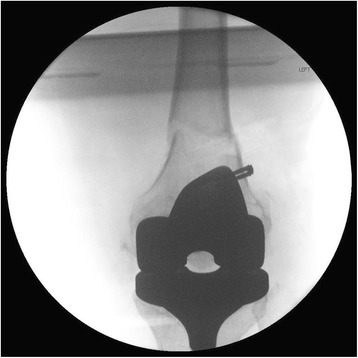
Intraoperative view of left femoral periprosthetic fracture after reduction and staple removal

**Fig. 4:**
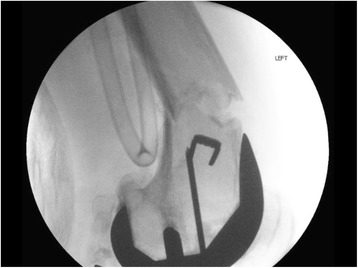
Intraoperative lateral view of left femoral periprosthetic fracture with guidewire placement

In our operating room a direct lateral approach through the previous incision was utilized to directly visualize the ACL staple. The staple was visible through the fracture site, and was removed easily (Fig. 3). The lateral femoral cortex was intact and had no significant cortical defects from the previous ACL tunnel. The total knee implant was then visualized using the original midline approach. After confirming the stability of the implant and reducing the fracture, cement in the intercondylar fossa was removed to establish the starting point for the intramedullary (IM) nail (Fig. 4). The previous ACL tunnel could not be identified. An 11 × 360 mm IM nail was inserted in a retrograde fashion with three locking screws placed distally and one proximally.

**Fig. 5:**
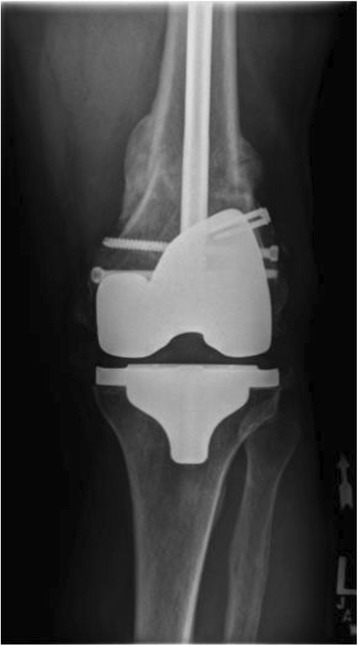
Anteroposterior view of the left femur after distal locking screw removal 5 months postoperatively

Postoperatively, our patient did well and achieved radiographic and clinical union after approximately 4 months. He underwent hardware removal of a symptomatic distal locking screw 5 months postoperatively without complication (Fig. 5). At the final 6-month follow-up, he was ambulating independently and back to preoperative functional capacity.

## Discussion

Distal femur fracture post-ACL reconstruction is a relatively rare complication on its own, and our literature search only identified 19 previous case reports describing this phenomenon. Fracture patterns in these case studies ranged from intra-articular femoral condyle fractures to supracondylar fractures. Patients with supracondylar fractures tended to have extra-articular points of ACL ligament fixation, which was in keeping with the results of our case study [1, 2, 9, 13, 16, 17, 19–21]. Only one case study demonstrated fracture after remote ACL reconstruction 11 years postoperatively, while most studies reported fractures within 1 to 2 years of the initial operation [2, 6]. Given the high frequency of ACL reconstructions and TKAs performed worldwide, we believe that previous ligamentous reconstructive surgeries may be an important adjunctive risk factor predisposing to TKA periprosthetic fractures.

A number of case studies identified in our literature search presented patients with distal femoral fractures due to stress riser effect from the bone tunnel or from complications related to bone tunnel graft, such as tunnel osteolysis [3, 8, 13, 14, 20]. Our case study differs from previous cases studies in that there was no visible cortical defect from the bone tunnel or intra-articular graft at the time of surgery, secondary to bony ingrowth and cement from the previous TKA procedure. In addition, the patient in our case study was significantly removed from his TKA and ACL operations (8 years and an estimated 25 years, respectively) compared to previous studies, allowing for further strengthening and remodeling of his metaphyseal femoral bone. Because the short oblique supracondylar fracture line originated distally and transverse to the implant, we hypothesize the periprosthetic fracture in this patient was primarily due to the stress riser effect of the ACL staple, and not sequela from the previous ACL tunnel, or from mechanical effects of his previous TKA.

Given this proposed mechanism of failure, we hypothesize that the ACL staples in our patient acted in a similar fashion to the effect of anterior femoral prosthesis notching in TKA. First, the supracondylar fracture pattern in our patient mimicked those seen in periprosthetic fractures with anterior cortical notching. Second, the location of the staple stress riser in our patient was at nearly the same distance as the most proximal aspect of the total knee prosthesis. As such, the force from any direct trauma to the knee would be concentrated at this bone–prosthesis interface, causing failure at the stress riser created by the near staple site. Although the cortical breech created by this staple may seem insignificant, biomechanical studies have shown that cortical defects created by femoral notching at this location can significantly weaken bending and torsional strength [4, 22, 23]. Torsional and bending strength are inversely related to the size of the cortical defect, with torsional strength more greatly compromised than bending strength. As such, most patients with anterior notching present with spiral or oblique periprosthetic fracture after low energy trauma, similar to our patient [4–22].

In a recent retrospective analysis of the Scottish joint registry, it was determined the most significant risk factors for periprosthetic fracture after arthroplasty included female gender, age (>70), and revision arthroplasty [24]. The patient in our study did not have any significant risk factors given that he was a young man with a primary TKA. However, other risk factors that have been reported in the literature include osteoporosis, rheumatoid arthritis, steroid therapy, neurological disease, cemented implants, cortical stress risers, and local osteolysis and infection [25, 26]. Often, patients have numerous risk factors, resulting in multifactorial etiology for such fractures. Other factors that could have contributed to the fracture in our patient may include osteoporosis and ACL tunnel osteolysis. Given his medical comorbidities, including diabetes and hypertension, and thin radiographic cortices, our patient may have been physiologically older, resulting in relative osteoporosis for his age. Unfortunately the patient in our study did not have any quantitative osteoporosis index studies completed. We also did not have any cross-sectional imaging or pre-fracture X-rays available to us and, as such, were unable to determine if there was any tunnel osteolysis from his previous ACL reconstruction. However, intraoperatively we were able to determine that there were no cortical defects related to his previous ACL tunnel.

Despite the rarity of distal femoral fracture after ACL reconstruction, this can be a devastating complication, which is only amplified in the setting of TKA. Revision surgery is associated with significant morbidity, which includes bone loss, infection, and implant instability. This case study demonstrates that remote history of ACL reconstruction can be an important risk factor to consider for periprosthetic fracture, even in a well-established and optimally fitted knee prosthesis. Fortunately, the patient in our case study did not require an extensive knee revision, as the prosthesis was stable, allowing us to insert a load-sharing IM device.

Current literature does not support the routine removal of hardware, but it is important to note that clinical outcomes from retained hardware have shown mixed results [27]. Theoretically, hardware removal can also create a stress riser effect leading to higher risk of fracture or other significant complications such as neurovascular injury [28]. Stress risers created from hardware removal should be ideally bypassed with stable fixation or protected with limited weight bearing transiently to avoid these complications. Hardware removal can be associated with significant complications, but excellent short-term results have also been reported in the literature [27, 29]. In this setting we chose to bypass the fracture and retained ACL staple with an IM nail in order to avoid further surgical dissection. Due to the infrequency of this complication, we cannot comment on whether ACL implants should be removed or used prior to TKA, but this should be determined on a patient-specific basis.

## Conclusions

Distal femoral fracture is a rare, but significant, postoperative complication of ACL reconstructive surgery. However, there has not yet been a reported case of periprosthetic fracture in well-established TKA associated with a remote ACL repair. Based on these findings, it may be important to consider removal of previous ACL reconstructive hardware prior to TKA to reduce risk of periprosthetic fracture, which should be determined on a patient-specific basis.
